# Patients Lacking the Capacity to Consent to Hip Fracture Surgery May Be Undergoing Major Operations Without Their Next of Kin Being Involved in Best-Interests Decisions: A Quality Improvement Report

**DOI:** 10.7759/cureus.20322

**Published:** 2021-12-10

**Authors:** Pardis Zalmay, Justin Collis, Helen Wilson

**Affiliations:** 1 Trauma and Orthopaedics, Royal Surrey County Hospital NHS Foundation Trust, London, GBR; 2 Trauma and Orthopaedics, Medway Maritime NHS Foundation Trust, Gillingham, GBR; 3 Geriatrics, Royal Surrey County Hospital NHS Foundation Trust, London, GBR

**Keywords:** orthogeriatric, orthopaedic, communication, mental cognition, consent form four, next of kin, hip fracture, patient consent, uk mental capacity act

## Abstract

Background

Cognitively impaired patients with a hip fracture may be undergoing major operations without attempts being made to involve their next of kin (NoK) in best-interest decisions.

Methods

We used the Plan-Do-Study-Act (PDSA) methodology to guide our quality improvement (QI) project. Cognitively impaired hip fracture patients were identified retrospectively by searching the hip fracture database of a medium-sized district general hospital (DGH). Their medical notes were reviewed for documented attempts at contacting their NoK prior to surgery as well as on completion of the NoK section of the Consent Form Four.

Intervention

A simple feedback intervention was delivered in the form of a mixed verbal and visual presentation to the orthopaedic registrars responsible for obtaining consent from these patients.

Results

Post-intervention, there were documented attempts at contacting the NoK before surgery for all patients, a significant improvement from only 80%. There was also a significant increase in completion of the NoK section of the consent form, from 30% to 64.3%.

Conclusions

Simple audit and feedback interventions can produce significant positive changes in communication between clinicians and the NoK of cognitively impaired patients with hip fractures. Further interventions have been implemented to sustain these improvements.

## Introduction

The National Health Service (NHS), as with all health systems in the world, is undergoing a transformation in its patient demographic. The ageing of the world population, contrary to previous beliefs, does not appear to be slowing down [1]. It is predicted that the proportion of the world population over 60 years of age will nearly double from 12% in 2015 to 22% in 2050 [2]. Given that musculoskeletal disorders are among the most common problems affecting older people, with osteoporosis at the forefront, the burden of fragility fractures is set to rise [3]. Hip fractures are one of the most commonly reported fragility fractures in older people [4].

According to conservative estimates, the annual number of hip fractures is set to rise from 1.66 million in 1990 to 6.26 million in 2050 worldwide [5]. Within the medical profession, it is well-understood that a hip fracture is a life-changing injury. The high rates of perioperative morbidity and mortality have contributed to the birth of the subspecialty of orthogeriatrics [6]. However, the public does not seem to share the clinicians’ appreciation of the severity of a hip fracture. Lay members of the public significantly underestimate the average hospital length of stay, and mortality associated with a hip fracture, even when they themselves or a relative have suffered from a hip fracture [7]. These findings hint at shortcomings in communication between healthcare professionals and the public.

This communication is vital when managing patients with cognitive impairment, as they often lack the ability to brief their next of kin (NoK) themselves. The Consent Form Four (CF) is filled out when treatment is offered to a patient who is deemed to lack the capacity to be involved in this decision-making. Within it, a section entitled ‘Involvement of those close to the patient’ outlines that the clinician taking consent should seek the views of those close to the patient, and requires them to document this discussion (Appendix 1]. This is part of the best-interests decision-making process as outlined by the Mental Capacity Act 2005 [8].

This quality improvement (QI) project was undertaken to investigate and improve preoperation communication between clinicians and the NoK of patients with hip fractures who lacked the capacity to consent to surgery.

## Materials and methods

Setting

This QI project was performed at a 520-bed district general hospital (DGH) in the UK that admits 260-340 patients with hip fractures annually [9]. The trauma unit provides specialist services in orthopaedic surgery and orthogeriatrics.

Intervention design and implementation

In practice, obtaining consent from patients admitted with hip fractures is a task undertaken by the on-call orthopaedic registrar, who can discuss the risks and intended benefits of the proposed procedure. When a patient is judged to lack the capacity to consent to a procedure, the Mental Capacity Act outlines that where appropriate, the clinician should seek the views of those interested in their welfare, usually referring to their NoK or lasting power of attorney [8]. There is no out-of-hours orthogeriatrics service, and patients are not always seen by an orthogeriatrician prior to going into surgery. Owing to these factors, orthopaedic registrars were selected as the targets of the intervention.

Healthcare professionals have been proven to be limited in their ability to accurately assess their own performance [10]. Audit and feedback refer to the practice of providing the auditee with a summary of their performance over a period of time [11]. Accurate feedback on how they perform compared to agreed standards can be an important motivator for change [12]. A Cochrane review found that feedback interventions can deliver modest but significant positive changes, particularly when baseline performance is low, feedback is provided more than once and is delivered in both verbal and written formats [11].

Drawing on these concepts, we implemented a two-pronged feedback intervention in the form of a verbal and visual presentation as well as a written email circulated to all clinical members of the orthopaedics team. The presentation was delivered by a foundation year two (FY2) doctor working within the orthopaedics department, at a trauma meeting attended by consultants, registrars, and junior trainees. After the presentation of the department’s performance, with care being given to not come across as critical of the orthopaedic trainees, the audience was invited to participate in the discussion. A consensus was reached that in the context of a patient deemed to lack capacity, it is the responsibility of the surgeon taking consent to contact the patient’s NoK.

Measures

Our primary outcome measure was documentation of attempts to contact the patient’s NoK prior to surgery. Our secondary outcome measure was the completion of the NoK section in the consent form.

Study population

**Figure 1:**
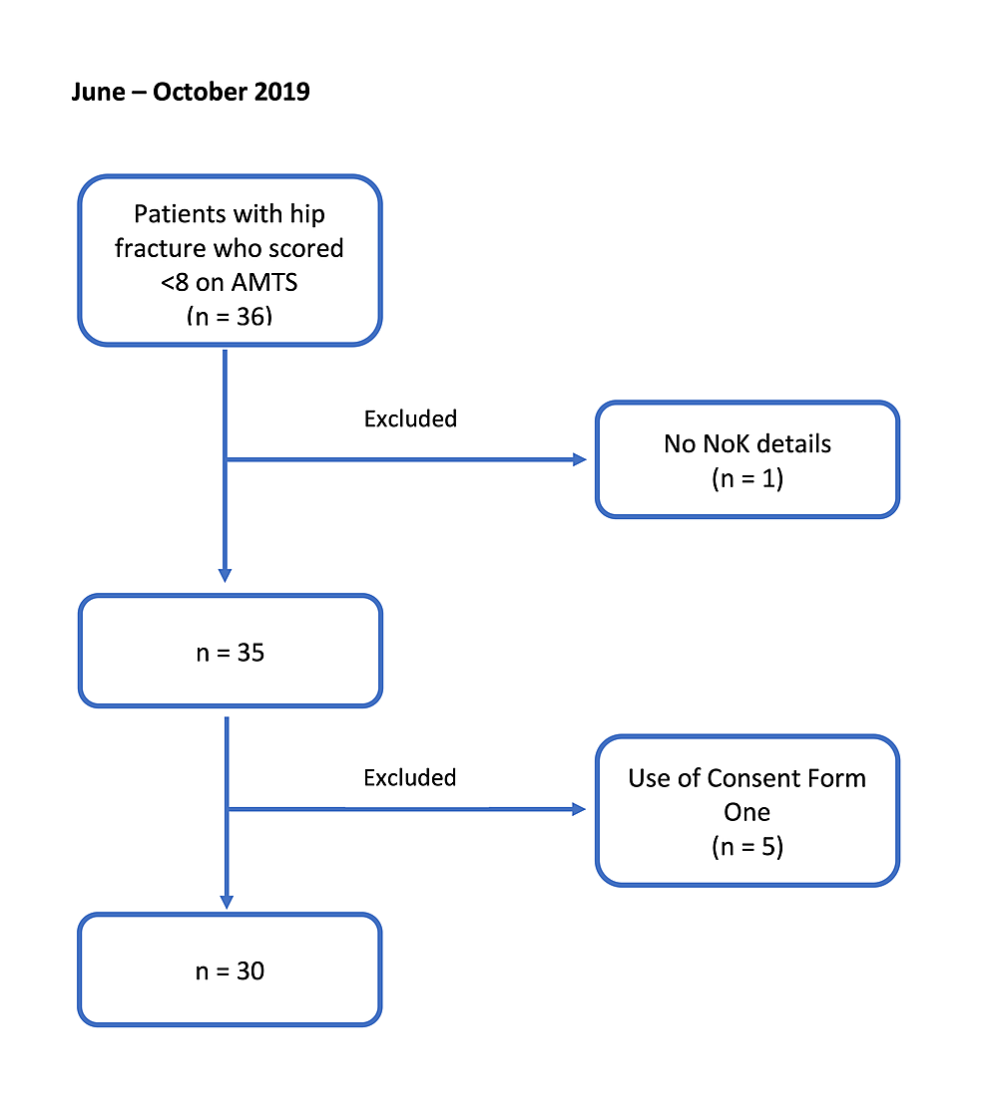
Patient selection criteria, June to October 2019 (pre-intervention) AMTS = Abbreviated mental test score; NoK = Next of kin

The Hip Fracture Database of the hospital was filtered to reveal patients admitted with a hip fracture between June and October 2019 who had an Abbreviated Mental Test Score (AMTS) of less than eight. Patients who had no next of kin and those deemed to have the capacity to consent were excluded, resulting in a total sample size of 30. Following the intervention, the same inclusion and exclusion criteria were used to identify patients admitted between May and September 2020, resulting in a sample size of 28. Figure 1 shows the patient selection criteria.

Analysis

Using the SPSS statistical software (IBM Corp. Armonk, NY), chi-square testing was utilized to test for statistical significance in the changes between pre-intervention and post-intervention outcomes.

Guiding future intervention

Following the second cycle of data collection, a semi-structured survey was delivered in person to the nine orthopaedic registrars involved in the on-call rota. Six survey items were forced-choice statements that the respondents were asked to rate their agreement with, on a scale of one (strongly disagree) to 10 (strongly agree). The final item was a ‘free-response’ invitation for suggestions on improving future performance (Appendix 2).

## Results

Pre-intervention, 80% (24/30) were noted to have had documented attempts at contacting their NoK prior to surgery. The section of Consent Form Four regarding the NoK discussion was completed in 30% (10/30).

Following the intervention, all 28 patients had documented attempts at contacting their NoK prior to going for hip surgery, a significant improvement (p <0.05). The Consent Form Four NoK section was appropriately completed in 64.3% (18/28) (p <0.05).

Survey results

**Figure 2:**
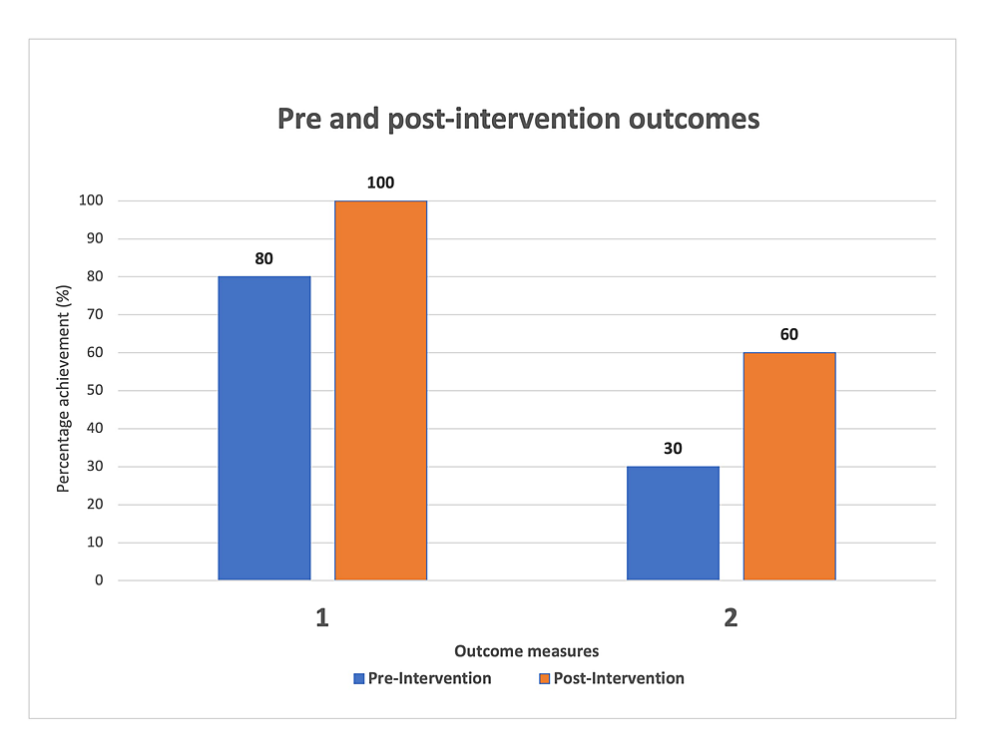
Comparison of results Outcome Measure 1 - Documented attempt to contact next of kin Outcome Measure 2 - Completion of next of kin section of the consent form

All nine of the orthopaedic registrars rostered on the on-call rota completed the survey; four provided an answer to the optional free-response item seven. Figure 2 shows a comparison of the results.

## Discussion

The aim of this project was to assess and improve the initial communication between the clinicians and the NoK of patients admitted with hip fractures. Following the intervention, a marked improvement was seen in the primary outcome measure. All 28 patients had documented attempts at contacting their NoK prior to going into surgery. There was also a significant improvement in the completion of the NoK section of the consent form.

The motivation for this project came about following a serious incident where a patient admitted with a hip fracture became gravely unwell on the operating table. Upon contacting the patient’s next of kin, they were surprised to hear that their relative was undergoing an operation. This project was founded on the belief that no patient should go into a potentially life-threatening operation without their next of kin being made aware. Compos mentis patients usually have the capacity to inform their families of their situation, but this is not always the case when cognition is impaired. The risk of mortality within the first 48 hours of hip fracture surgery has been estimated at 0.8%, erring close to the threshold where death can be considered a ‘common’ event [13]. Outside of the immediate post-operative period, estimates put the rate of mortality at 17%-38% at the end of the first year [14]. This is not to mention that patients with cognitive impairment are at a higher risk of mortality than those without [15].

As such, the finding that 20% of patients were possibly going into hip fracture surgery without the admitting team attempting to notify their NoK was regarded as unacceptable.

Surgeons, particularly orthopaedic surgeons, attract more complaints than their medical peers. Though some of this has to do with the nature of surgical procedures, a significant portion of this is related to interpersonal skills and communication [16-17]. Qualitative studies investigating the experiences of families of patients with hip fracture reveal that families are surprised by the lack of communication from healthcare providers in the early days of admission. Family members report having to be persistent and proactive in order to receive updates on their loved ones [18-19]. In their review looking into legal claims made to the NHS Litigation Authority regarding patients with hip fractures, Ring et al. stressed that more efforts need to be made to involve NoK in discussions so they understand the risks involved with the injury [20].

The advent of the orthogeriatrics subspecialty has undoubtedly led to reduced mortality and improved outcomes in patients admitted with hip fracture [21-22]. With the well-evidenced push for prompt surgery, it is important that orthopaedic surgeons are comfortable having early discussions with the families of cognitively impaired patients [23]. This is one aspect of the ­patient’s journey that the orthopaedic surgeon should become comfortable with.

Key findings of the survey

Item one: Encouragingly, all respondents answered ten, suggesting that the orthopaedic registrars were aware of the importance of communicating with NoK.

Items three and four: The heterogeneity of responses to these items suggested possible room for improvement in the design of the Consent Form Four.

Item six: On answering whether they enjoyed discussions with NoK, responses demonstrated significant variance with a range of answers from one to ten. This provides potential insight into why performance in the secondary outcomes remained low despite the respondents reportedly understanding the importance of communication with NoK.

Item seven: Once again, heterogeneity in attitudes towards the Consent Form Four was demonstrated here.

Table 1 lists the results of the survey.

**Table 1:** Orthopaedic registrar survey results (Appendix 2) Left column = Survey items. Top row = Training grades. NoK = Next of kin; CCT = Certificate of completion of training; ST = Specialty trainee; T&O = Trauma & orthopaedics; SpR = Specialist registrar; SHO = Senior house officer; C4 = Consent Form Four

­­­­Item	Post CCT Fellow	ST5	ST3	Fellow	ST7	Non-Trainee	Registrar	ST7	ST4	Average
1	10	10	10	10	10	10	10	10	10	10
2	9	5	10	10	10	10	10	10	10	9.33
3	7	10	10	10	3	7	1	10	10	7.56
4	9	5	8	1	8	10	10	3	5	6.56
5	10	10	10	10	7	10	10	10	10	9.67
6	8	6	6	7	3	10	5	1	6	5.78
7	The section can get "lost" in all the text of form 4. A more clear "aide-memoire" would help.	E-training package for T&O + orthogeris SpR/SHO Blank space on C4 usefully ambiguous to record individual's discussion. We just need to use the space better.		Form 4 is fine.			Most conversations on the telephone with NoK and the space for those discussions is too small.			

Implications

The UK Foundation Programme Curriculum requires foundation doctors to demonstrate involvement in clinical audits or a quality improvement (QI) project in order to progress through their training. Research has shown that only around half of foundation year-one doctors can demonstrate completion of an audit, with cited barriers being the selection of audits that are too large or unmanageable, poor support from senior staff, and lack of time [24]. This project supports previous research that shows that careful selection, audit and feedback can be a cost-effective and time-efficient means of effecting clinically useful change [10].

Limitations

A major limitation of this project was that we measured the documentation of attempts to contact next of kin; the actual rates of clinicians contacting next of kin may have been higher. Leading on from this, the improvements we observed may have reflected an improvement in documentation rather than actual communication. Annual surgical rotations occur in October in the UK; the intervention and second cycle of data collection were performed on a slightly different cohort of orthopaedic registrars than the first cycle of data collection. Thus, the improvements seen may have been related to individual differences within the cohort. Further, any positive effects related to the feedback intervention are likely to diminish, as some of that cohort of registrars rotate to a different hospital. In order to ensure all the orthopaedic registrars completed the survey, they were approached in person. This lack of anonymity may have led to social desirability bias affecting the survey responses. The sample size of the survey was small so any conclusions drawn are not generalizable.

Further interventions

The planned future interventions are the timing of repeat audit loops to occur at the annual change-over of orthopaedic registrars; consent form e-training modules to be completed as part of the induction process for surgical trainees rotating into the Trust; re-design of the Consent Form Four to highlight the mandatory nature of discussions with NoK, with sufficient room to document reasons for discussions not happening if deemed inappropriate.

## Conclusions

Cognitively impaired patients lacking the capacity to consent to surgery for hip fractures may be undergoing major surgical procedures without their next of kin involved in best-interest decisions. This project identified potential shortcomings in communication, and following a relatively simple feedback intervention, was able to record marked improvements in this outcome measure.

## Figures and Tables

**Figure 3:**
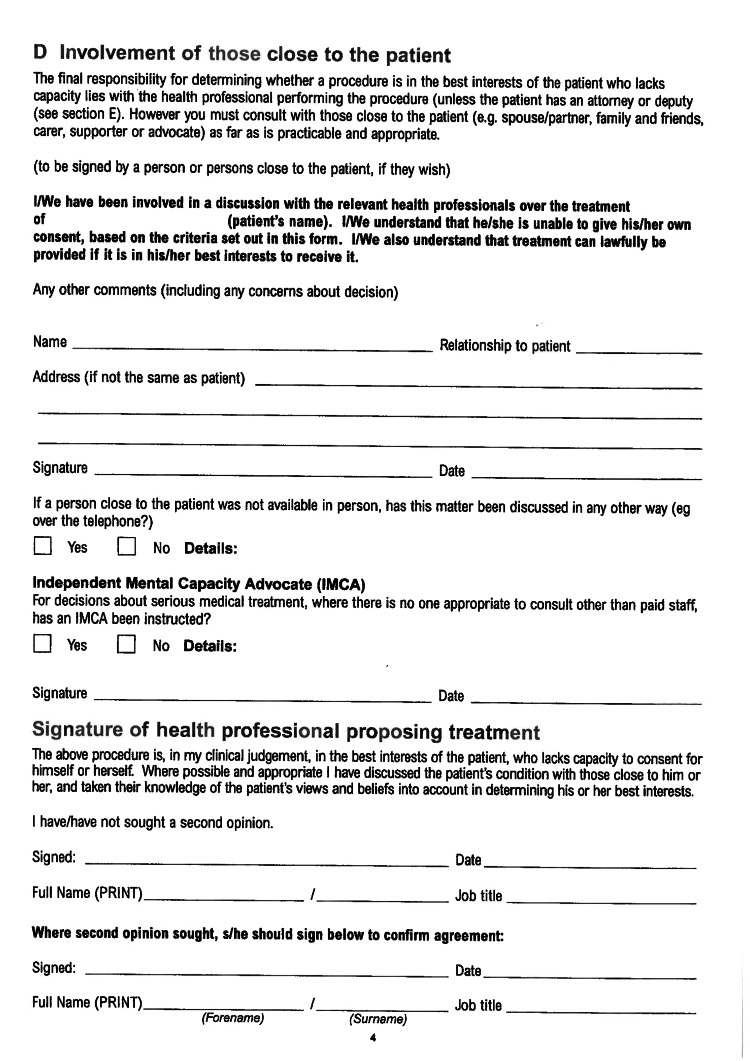
Appendix 1

**Figure 4:**
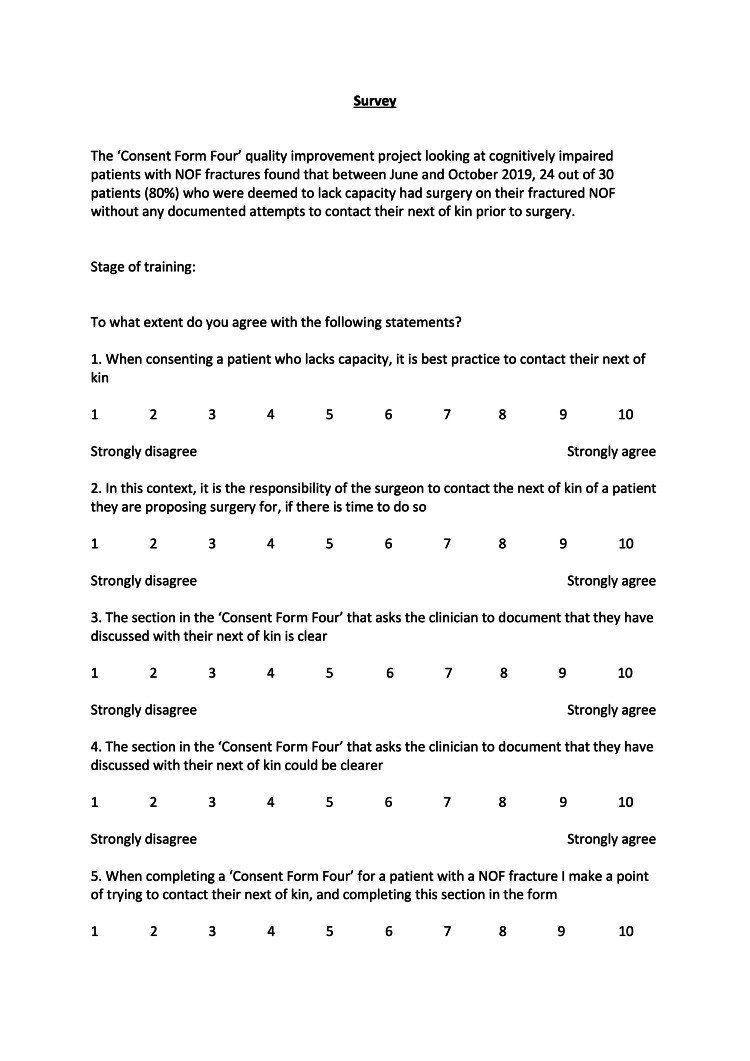
Appendix 2
